# GLUD1 suppresses renal tumorigenesis and development *via* inhibiting PI3K/Akt/mTOR pathway

**DOI:** 10.3389/fonc.2022.975517

**Published:** 2022-09-20

**Authors:** Lei Wang, Zhiyu Fang, Peixiang Gao, Junfang Zheng

**Affiliations:** ^1^ Department of Urology, Beijing Friendship Hospital, Capital Medical University, Beijing, China; ^2^ Beijing Key Laboratory of Cancer Invasion and Metastasis Research, Department of Biochemistry and Molecular Biology, School of Basic Medical Sciences, Capital Medical University, Beijing, China

**Keywords:** GLUD1, renal cell carcinoma, prognosis, methylation, PI3K/Akt/mTOR

## Abstract

Growing cancer cells are addicted to glutamine. Glutamate dehydrogenase 1 (GLUD1) is one of key enzymes in glutamine metabolism and plays a critical role in the malignancy of diverse tumors. However, its role and molecular mechanism in clear cell renal cell carcinoma (ccRCC) development and progression remain unknown. In this study, analysis results of the GEO/TCGA/UALCAN database showed that GLUD1 level was downregulated in ccRCC tissues. Immunohistochemistry and western blotting results further validated the downregulation of GLUD1 level in ccRCC tissues. GLUD1 level was gradually decreased as ccRCC stage and grade progressed. Low GLUD1 level was associated with a shorter survival and higher IC50 value for tyrosine kinase inhibitors (TKIs) in ccRCC, reminding that GLUD1 level could predict the prognosis and TKIs sensitivity of ccRCC patients. High level of methylation in *GLUD1* promoter was positively correlated with the downregulation of GLUD1 level and was negatively correlated with survival of ccRCC patients. GLUD1 overexpression suppressed RCC cell proliferation, colony formation and migration by inhibiting PI3K/Akt/mTOR pathway activation. Low GLUD1 level correlated with suppressive immune microenvironment (TIME) in ccRCC. Together, we found a novel tumor-suppressing role of GLUD1 in ccRCC which was different from that in other tumors and a new mechanism for inhibiting PI3K/Akt/mTOR activation and TIME in ccRCC. These results provide a theoretical basis for GLUD1 as a therapeutic target and prognostic marker in ccRCC.

## Introduction

More than 400,000 new cases of renal cell carcinoma (RCC) and nearly 180,000 deaths occurred each year (1). Clear cell renal cell carcinoma (ccRCC) is the most common and invasive subtypes of RCC type (2). The prognosis of ccRCC has been drastically improved over the past decades with the emergence of tumor immunotherapy (3). Currently, most patients are treated with immune checkpoint inhibitors (ICIs) or a combination of ICIs and tyrosine kinase inhibitors (TKIs). Despite clear benefit of these treatments over large populations, there are still some patients who are innately resistant to these treatments or develop resistance within a few months (4). Therefore, there is an urgent need to develop predictive biomarkers that can guide treatment strategies at the individual level.

Glucose and glutamine are the two main nutrient sources during cell proliferation. ccRCC is addicted to glutamine (5–8). Glutamine is converted to glutamate by glutaminase (GLS or GLS2) (9). Glutamate is converted to α-ketoglutarate (α-KG) through one of two sets of enzymes, glutamate dehydrogenase 1 (GLUD1) or transaminase (10). Then, α-KG enters the tricarboxylic acid (TCA) cycle and generates ATP (11). Through the TCA cycle, glutamate also provides precursors for the biosynthesis of amino acids, nucleotides, lipids and reducing equivalents in the form of NADH (necessary for oxidative phosphorylation to synthesize ATP) and NADPH (required for lipid and nucleotide biosynthesis). Since GLUD1 regulated glutaminolysis, ATP production, biosynthesis and affected the occurrence and progression of breast cancer, gastric cancer and lung cancer (12–14), and highly proliferative human tumors display high transaminase and low GLUD expression (14). Therefore, GLUD1 might play a crucial role in ccRCC development and progression. Currently, the role and mechanism of GLUD1 in ccRCC occurrence and development remains unknown.

In this study, we found that GLUD1 expression level was downregulated in ccRCC tissues. Downregulated GLUD1 level was correlated with ccRCC malignancy, and poor prognosis and TKIs sensitivity. The increased methylation in *GLUD1* promoter led to the downregulation of GLUD1 level. GLUD1 suppressed ccRCC cell proliferation, colony formation and migration by inhibiting PI3K/Akt/mammalian target of rapamycin (mTOR) pathway activation. *GLUD1* level was negatively associated with the tumor immunosuppressive microenvironment (TIME). GLUD1 might be a novel prognostic, predictive marker and potential therapeutic target for ccRCC.

## Materials and methods

### Bioinformatics analyses

The microarray series (GSE53757) information was obtained from the National Center for Biotechnology Information Gene Expression Omnibus database (NCBI GEO, https://www.ncbi.nlm.nih.gov/gds/?term=GSE53757). The TCGA *GLUD1* mRNA level data (RNA Seq v2) in RCC patients was from https://www.synapse.org/. The clinical data were from cBioPortal database (www.cbioportal.org). The protein levels of GLUD1 were from the UALCAN database (http://ualcan.path.uab.edu/).

### Tissue collection

Surgical specimens of ccRCC cases and adjacent normal renal tissues were collected from nephrectomy specimens at the Affiliated Beijing Friendship Hospital, Capital Medical University in December 2017. 10 paired ccRCC and normal renal specimens were formalin-fixed and paraffin-embedded for immunohistochemistry (IHC) analysis. Another 12 paired fresh samples were immediately frozen in liquid nitrogen and stored at -80°C for use in western blotting (WB) analysis. All specimens were histologically confirmed by uro-pathologists. The study was approved by the Research Ethics Board of Affiliated Beijing Friendship Hospital and was performed according to the World Medical Association Declaration of Helsinki. All subjects included in the protocol signed a declaration of informed consent. Prior to surgery, the patients had not received any therapies.

### Immunohistochemistry

IHC was performed as described before (15). The sections were incubated with anti‐GLUD1 antibody (Cat# PTM-5632, 1:100, PTM Biolabs Inc., Hangzhou, China). Image‐Pro plus 6.0 (MediaCybernetics Inc., SilverSpring, MD, USA) was used to analyze optical densitometry.

### Western blotting

WB was performed as previously described (16). The primary antibodies comprised anti-Flag (Cat# AE063), anti-Akt (Cat# A17909), anti-p-Akt (Cat# AP1259), anti-mTOR (Cat# A11354), anti-p-mTOR (Cat# AP0978), anti-GAPDH (Cat# A19056) (all from Abcam, Cambridge, UK) and anti‐GLUD1 antibody (Cat# PTM-5632, PTM Biolabs Inc). The secondary antibodies comprised HPR-labeled anti-rabbit antibody (Cat# ZB-2301) and HPR-labeled anti-mouse antibody (Cat# ZB-2305) (all from ZSGB-BIO, Beijing, China).

### Gene set enrichment analysis

The association between phenotypes and *GLUD1* expression level was analyzed using gene set enrichment analysis (GSEA v2.2, http://www.broad.mit.edu/gsea/) as previously described (17). A gene set is considered significantly enriched when the false discovery rate (FDR) score is < 0.05.

### Methylation level analysis of GLUD1 promoter and correlation analyses with phenotypes and survival

UALCAN online tool (http://ualcan.path.uab.edu/cgi-bin/ualcan-res.pl) was used to analyze methylation levels of *GLUD1* promoter in ccRCC and paracancerous tissues. The correlation between methylation levels of *GLUD1* promoter and *GLUD1* mRNA level and correlation between the methylation level of *GLUD1* promoter and clinical phenotype were analyzed by MEXPRESS tool (https://mexpress.be/index.html). MethSurv was used to perform multivariable survival analysis using DNA methylation data.

### Plasmid construction, cell culture and transfection

The human renal carcinoma cell lines ACHN and 769-P were obtained from American Type Culture Collection and cultured according to the standard protocols. ACHN and 769-P cells were cultured in RPMI-1640 medium, containing fetal bovine serum (FBS) at a final concentration of 10%. All cell culture reagents were provided by HyClone (Logan, UT, USA). Lipofectamine 2000 (Invitrogen Carlsbad, CA, USA) was used for GLUD1-Flag (Zeqiong, Changsha, China) transfection according to the manufacturer’s protocol.

### Cell proliferation assay

The Cell Counting Kit-8 (Dojindo, Kumamoto, Japan) colorimetric assay was conducted to measure the relative number of viable cells (18).

### Colony formation assay

The single cell colony formation abilities were measured by plate colony assay in a 6-well plate (19). Triplicate experiments with triplicate samples were performed.

### Wound healing assay

Cells were seeded into 6-well cell culture plate and cells were wounded with the tip of a P-20 microtube. Then, wound healing was monitored and measured.

### Co-expression gene network of GLUD1

Co-expression online analysis was performed in the website (https://www.cbioportal.org/) by using the mRNA level in the TCGA_KIRC database (TCGA, Nature 2013). With *P* value < 0.05 as the threshold, the genes which had greater than 0.3 Spearman correlation coefficient with *GLUD1* in expression level were selected. Cytoscape software (Cytoscape_v3.8.0) was used to draw gene co-expression network.

### Protein-protein interaction (PPI) network construction of GLUD1-related genes

GEO2R (http://www.ncbi.nlm.nih.gov/geo/geo2r/) was applied to uncover differentially expressed genes (DEGs) between ccRCC tumors and adjacent renal tissues. DEGs were screened out using GEO2R according to the criteria of *P* value < 0.05 and |logFC| > 1. The DEGs which were statistically correlated with GLUD1 (|spearman coefficien| > 0.3) were defined as GLUD1-related genes.

Search Tool for the Retrieval of Interacting Genes/Proteins (STRING, http://string-db.org/) is a database used to predict the interaction among GLUD1-related DEGs. The minimum interaction value is set to 0.4 (medium confidence), and protein nodes that do not interact with other proteins are removed. Then, the network graph was visualized and analyzed using Cytoscape v3.8.0.

### KEGG pathway analysis

KEGG analysis was executed by online analysis tools–Database for Annotation, Visualization, and Integrated Discovery (DAVID) (http://david.abcc.ncifcrf.gov/).

### Analyses of the correlations of GLUD1 levels with the immunosuppressive microenvironment of ccRCC

The correlations of *GLUD1* level with immunosuppressive cells abundances were analyzed on the TISIDB database (http://cis.hku.hk/TISIDB/index.php). Immune score was estimated using Sangerbox (http://vip.sangerbox.com/home.html) for assessing the association between tumor microenvironment components and *GLUD1* expression in ccRCC. Tumor Immune Dysfunction and Exclusion (TIDE) (http://tide.dfci.harvard.edu) computational methods were used to predict T cell dysfunction.

### Statistical analysis

Statistical analyses were performed using IBM SPSS 26 (SPSS, Inc., Chicago, IL, USA) and Graphpad Prism 8 (Graphpad Software, Inc., San Diego, CA, USA). The paired samples were analyzed by paired samples *t*-test. The unpaired samples were analyzed by independent samples *t*-test. The relationship between GLUD1 expression level and clinical stages was analyzed by one-way ANOVA. Overall survival analysis was evaluated by Kaplan–Meier plots and log-rank tests. Correlation between methylation levels of *GLUD1* promoter and *GLUD1* mRNA level was analyzed by Pearson correlation analysis. Proliferation curve results were analyzed using a repeated measures ANOVA. Correlations between gene expression levels and among *GLUD1* level and immune cells infiltration were analyzed by Spearman correlation analysis. A *P*-value < 0.05 was deemed statistically significant.

## Results

### GLUD1 is downregulated in ccRCC tissues

**Figure 1:**
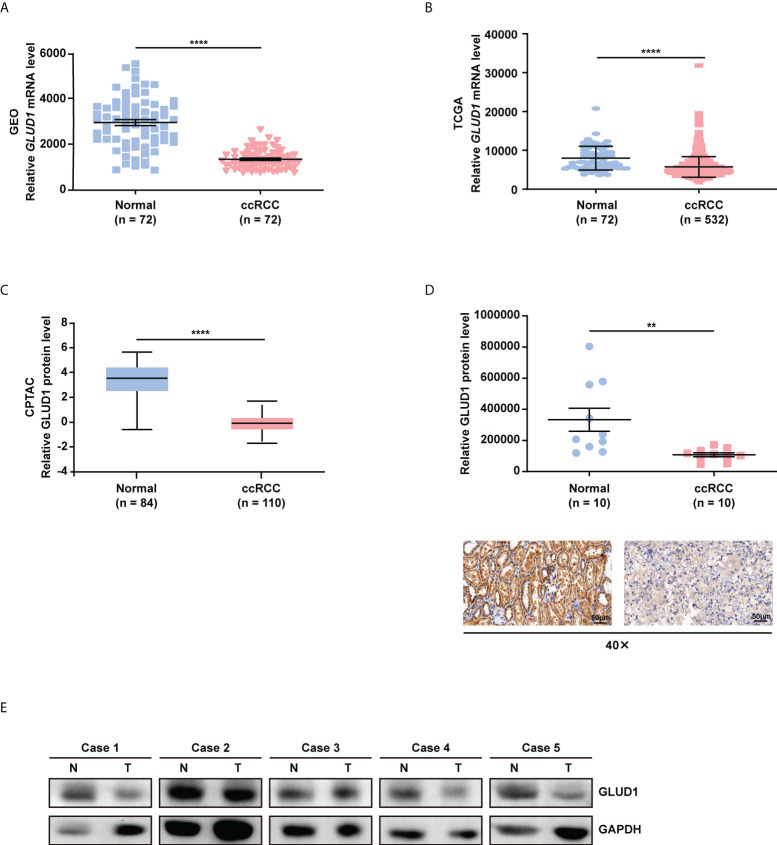
GLUD1 is downregulated in ccRCC tissues. **(A, B)** *GLUD1* mRNA levels in ccRCC tissues compared with normal tissues based on GEO GSE53757 data **(A)** and TCGA_KIRC data **(B)** respectively. **(C)** GLUD1 protein levels in ccRCC tissues compared with adjacent normal tissues based on CPTAC data. **(D)** GLUD1 protein levels in ccRCC tissues and normal tissues were detected using IHC. Scatter plot displaying the expression of GLUD1 in adjacent normal tissues and ccRCC tissues. **(E)** GLUD1 protein levels in ccRCC tissues and paired normal tissues were detected using western blot assay. **P < 0.01; ****P < 0.0001.

In order to clarify the role of GLUD1 in ccRCC, we analyzed the data from GEO database (GSE53757) and the TCGA_KIRC database. We found that *GLUD1* mRNA levels were significantly downregulated in ccRCC tissues compared with nontumor tissues (**Figures 1A, B**). Moreover, based on CPTAC database, we found that GLUD1 protein levels were also decreased in ccRCC tissues compared with normal renal tissues (**Figure 1C**). Subsequently, we examined GLUD1 protein levels in ccRCC tissues and adjacent renal tissues by IHC and WB, respectively. The results further confirmed that the protein level of GLUD1 in the ccRCC tissues was lower than that in the matched adjacent normal tissues (**Figures 1D, E**). All these results demonstrate that GLUD1 expression level is downregulated in ccRCC tissues.

### GLUD1 is a potential prognostic and TKIs sensitivity predictive markers for ccRCC patients

**Figure 2:**
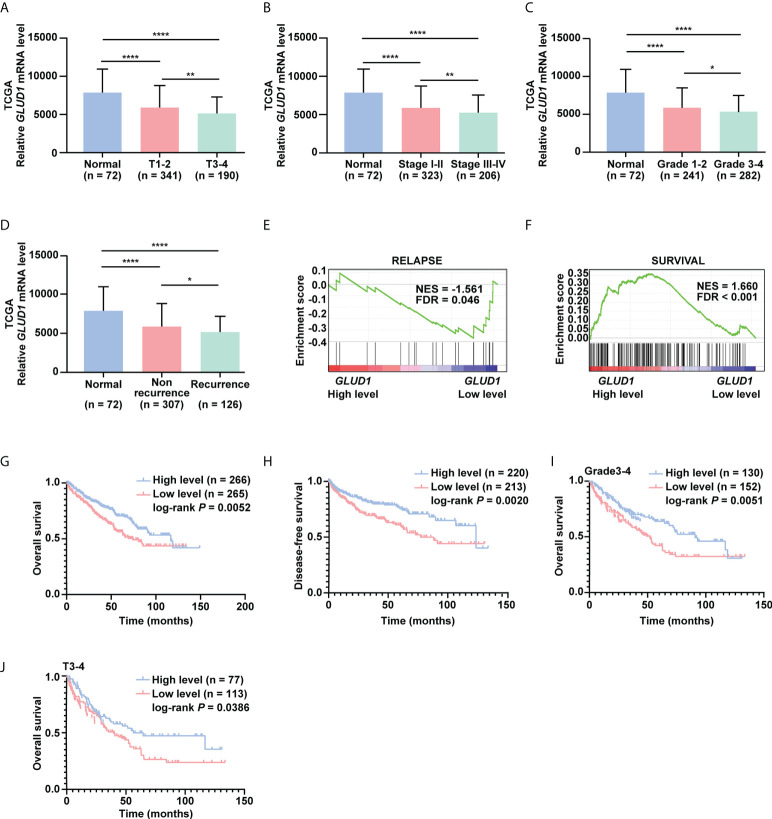
GLUD1 is a potential prognostic and TKI sensitivity predictive marker for ccRCC patients. **(A–C)** *GLUD1* mRNA levels were gradually downregulated as T stage, AJCC stage and Fuhrman grade progressed. **(D)** *GLUD1* mRNA level was negatively correlated with recurrence of ccRCC patients. **(E, F)** Enrichment plots of gene expression signature for relapse (SMID_BREAST_ CANCER_RELAPSE_IN_LUNG_UP) and survival (LEE_LIVER_CANCER_SURVIVAL_DN) were obtained by GSEA according to *GLUD1* mRNA levels. The ccRCC samples from TCGA_KIRC database were divided into high and low GLUD1 expression groups according to the median value of *GLUD1* RNA-seq quantification results. **(G, H)** The Kaplan-Meier (KM) curves of overall survival and disease-free survival based on TCGA_KIRC data. ccRCC patients were divided into high/low expression groups according to *GLUD1* mRNA level. **(I, J)** KM curves of overall survival based on TCGA_KIRC data. Advanced ccRCC patients were divided into high/low expression groups according to *GLUD1* mRNA level. *P < 0.05; **P < 0.01; ****P < 0.0001.

To investigate the clinical significance of GLUD1 downregulation in ccRCC tissues, we analyzed the correlation between GLUD1 expression level and clinicopathological characteristics. *GLUD1* mRNA level was gradually decreased as T stage, AJCC stage and Fuhrman grade progressed (**Figures 2A** **–C**). The low level of *GLUD1* was related with recurrence and relapse of ccRCC patients (**Figures 2D, E**), while high level of *GLUD1* was related with survival of ccRCC patients (**Figure 2F**). These results reveal that GLUD1 may be a tumor suppressor in ccRCC and potential prognostic marker for ccRCC patients.

To further investigate the clinical implications of GLUD1 downregulation in ccRCC, the correlation between the *GLUD1* mRNA level and survival rates of patients was analyzed based on the TCGA data. The results indicated that low *GLUD1* level predicted shorter overall survival (OS) and disease-free survival (DFS) (**Figures 2G, H**), especially for patients in higher Fuhrman stage and T stage (**Figures 2I, J**). In addition, we also observed high *GLUD1* expression was related with the higher sensitivity of ccRCC cells to TKIs treatment (**Figures S1A, B**). Collectively, these findings reveal that GLUD1 level is a potential prognostic and TKIs sensitivity predictive markers for ccRCC patients.

### High level of methylation in *GLUD1* promoter leads to the downregulation of GLUD1 level in ccRCC tissues and correlates with the survival of ccRCC patients

**Figure 3:**
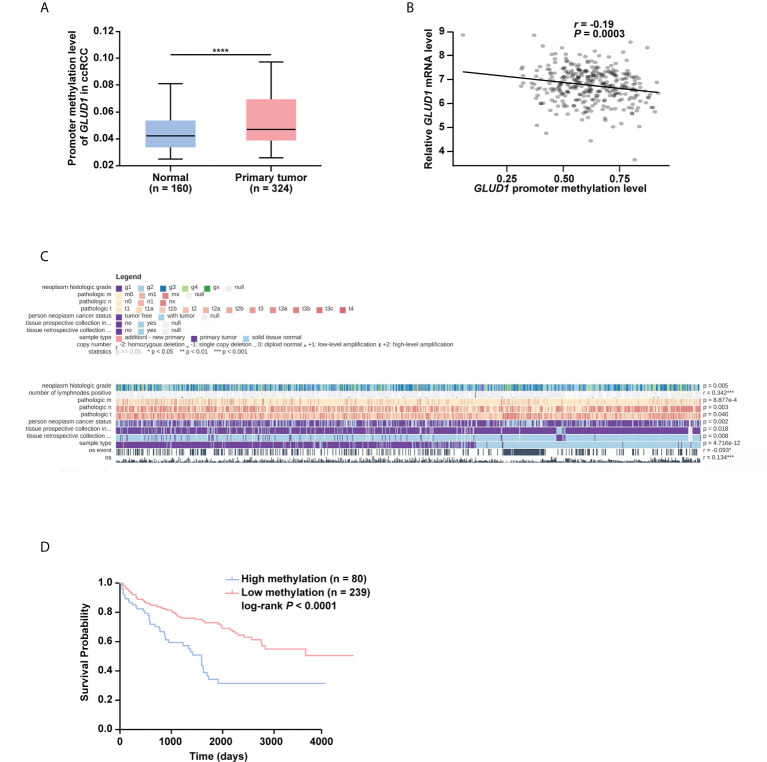
High level of methylation in *GLUD1* promoter leads to the downregulation of GLUD1 level in ccRCC tissues and correlates with shorter survival of ccRCC patients. **(A)** Methylation levels of *GLUD1* promoter was increased in ccRCC tissues based on the TCGA_KIRC data. The Beta value indicated the level of DNA methylation, and the *P* value was derived from independent sample two tailed *t*-test. The data were presented as mean ± SD. ****P < 0.0001. **(B)** The correlation between methylation levels of *GLUD1* promoter and *GLUD1* mRNA level. **(C)** The correlation between *GLUD1* methylation levels and clinical parameters in the MEXPRESS database. **(D)** KM curve of methylation levels of *GLUD1* promoter based on the TCGA data. ****P < 0.0001.

To further explore the underlying mechanism of GLUD1 downregulation, the bioinformatics analyses were performed. Results showed that *GLUD1* gene had low frequency in mutation (**Figure S2**), suggesting that decreased GLUD1 level in ccRCC tissues does not result from gene mutation. However, the methylation level of *GLUD1* promoter in ccRCC tissues was significantly increased (**Figure 3A**). In addition, correlation analysis results showed that the methylation level of *GLUD1* promoter was negatively correlated with the *GLUD1* mRNA level (**Figure 3B**). These results reminded that the increasing methylation level of *GLUD1* promoter might be one of the mechanisms for GLUD1 downregulation in ccRCC. Based on MEXPRESS database, we found that the methylation levels of *GLUD1* promoter in ccRCC tissues were significantly upregulated as neoplasm histologic grade progressed and positively correlated with lymph node metastasis (**Figure 3C**). We further found that the high level of methylation in *GLUD1* promoter was related with shorter OS of ccRCC patients (**Figure 3D**). These results suggest the methylation in *GLUD1* promoter has the important biological significance in ccRCC phenotypes.

Then, we explored which methylation-related writers and erasers were responsible for elevated methylation levels of *GLUD1* promoter. Results showed DNMT1, DNMT3A and DNMT3B were upregulated, while KDM1A was downregulated in ccRCC tissues (**Figures S3A–D**). Further Pearson correlation analysis revealed the most significant negative correlation of *GLUD1* level with DNMT3A in ccRCC tissues (*r* = -0.3806, *P* < 0.0001, **Figure S3E**). These results suggest that DNMT3A may play a crucial role in regulating the methylation level of *GLUD1* promoter in ccRCC.

### GLUD1 suppresses the proliferation and migration of RCC cells

**Figure 4:**
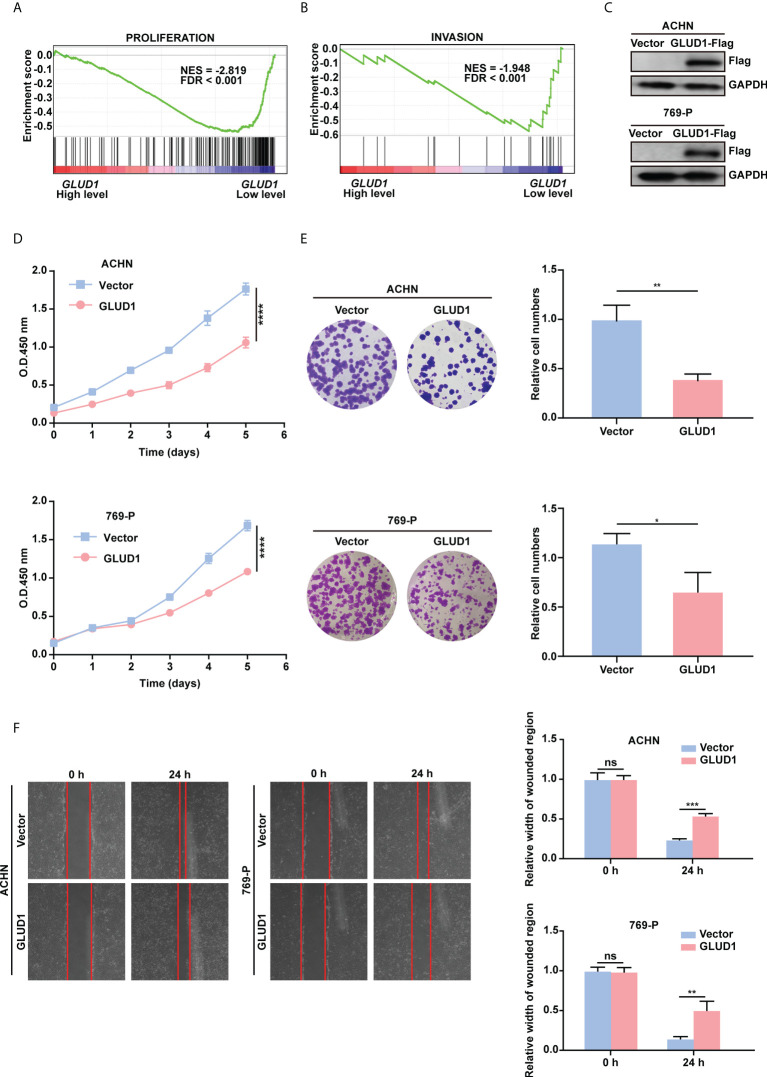
GLUD1 suppresses the proliferation and migration of RCC cells. **(A, B)** Enrichment plots of gene expression signature for proliferation (CHIANG_LIVER_CANCER_SUBCLASS_ PROLIFERATION_DN) and invasion (MINGUEZ_LIVER_CANCER_VASCULAR_INVASION_ DN) were obtained by GSEA according to *GLUD1* mRNA levels. The ccRCC samples from TCGA_KIRC database were divided into high and low GLUD1 expression groups according to the median value of *GLUD1* RNA-seq quantification results. **(C)** ACHN and 769-P cells were transfected with GLUD1-Flag expression plasmid, and protein levels were detected using western blot assay. β-actin was used as a loading control. **(D–F)** ACHN and 769-P cells were transfected with GLUD1-Flag. Vector-transfected ACHN and 769-P cells served as control. CCK8 viability assays were used to analyze cell viability **(D)**. Colony formation assays were performed to detect cell proliferation **(E)**. Wound healing assays were performed to detect cell migration **(F)**. The relative migration distance is quantified. ns, no significance; *P < 0.05; **P < 0.01; ***P < 0.001; ****P < 0.0001.

To examine the role of GLUD1 in RCC cells, GSEA was performed based on TCGA_KIRC data. Results revealed that gene sets associated with cell proliferation and invasion were significantly enriched in cases with low *GLUD1* expression (**Figures 4A, B**). These results suggest that the low expression of GLUD1 may promote ccRCC tumorigenesis and development. To validate the biological effects of GLUD1 in ccRCC, GLUD1 was overexpressed in ACHN and 769-P cells. GLUD1 overexpression significantly suppressed the proliferation, colony formation and migration of ACHN and 769-P cells (**Figures 4C–F**). In summary, these data indicate that GLUD1 suppresses RCC cell proliferation and migration.

### GLUD1 suppresses ccRCC tumorigenesis and development by inhibiting PI3K/Akt/mTOR pathway

**Figure 5:**
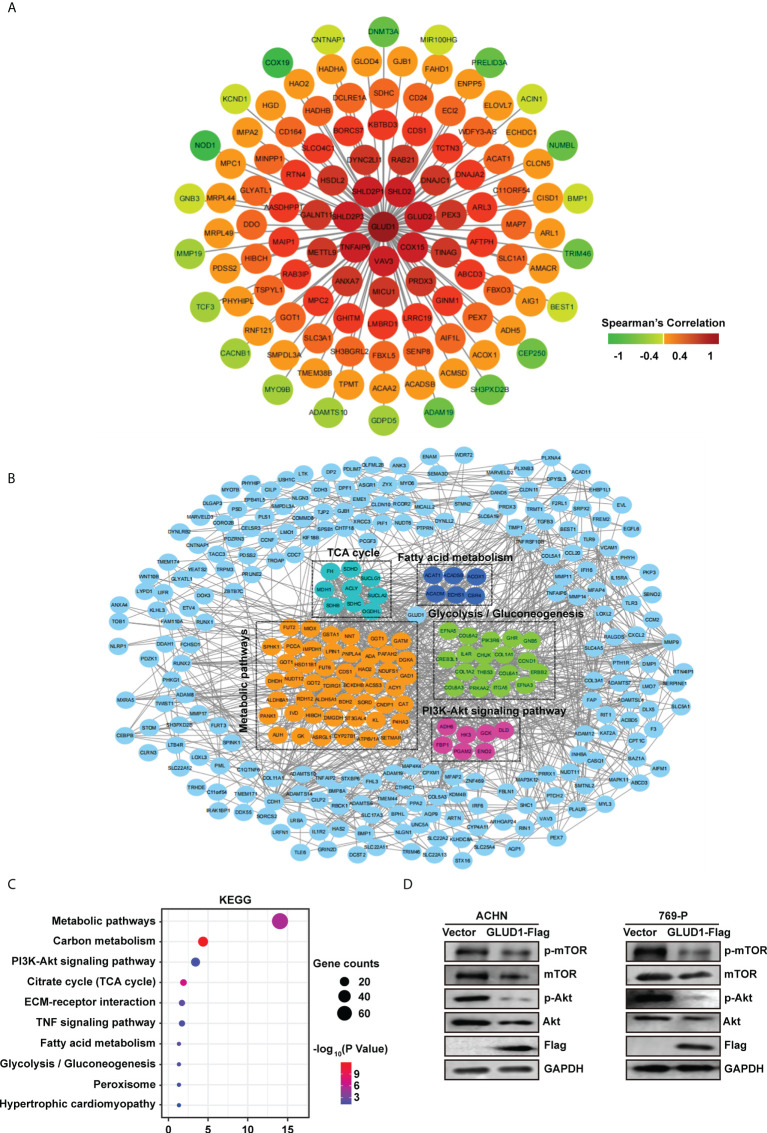
GLUD1 suppresses ccRCC tumorigenesis and development by inhibiting PI3K/Akt/mTOR pathway. **(A)** Co-expression network of *GLUD1* gene in ccRCC. Genes that were negatively correlated with GLUD1 were displayed in green, and genes that were positively correlated with GLUD1 were displayed in red. The darker the color, the stronger the correlation. **(B)** PPI network of GLUD1-related DEGs. **(C)** KEGG pathway enrichment analysis of GLUD1-related differentially expressed proteins. **(D)** Western blot measured the levels of proteins related to PI3K/AKT/mTOR pathway in ACHN and 769-P cells with GLUD1 overexpression.

To clarify the mechanism by which GLUD1 suppressed ccRCC occurrence and development, we analyzed the genes correlated with GLUD1 expression in TCGA dataset. Co-expressed genes can better clarify the role of GLUD1 in ccRCC. We constructed co-expressed gene network and found that GLUD2, COX15, VAV3, TNFAIP6, SHLD2P3, SHLD2P1, SHLD2, *etc.* were moderately correlated with GLUD1 (**Figure 5A**).

In order to elucidate the mechanism of GLUD1 in ccRCC more clearly, we chose the GLUD1-related proteins which were not only correlated with GLUD1, but also differentially expressed between ccRCC and adjacent normal tissues. GLUD1-related proteins were used to construct PPI network. We found that SDHD, ECSH1, COL6A3, CAT, ADH6, *etc.* were GLUD1-related proteins (**Figure 5B**).

Then, KEGG enrichment analysis was performed based on GLUD1-related proteins. Results showed that most of genes were enriched in PI3K/Akt/mTOR pathways (**Figure 5C**). The aberrant activation of PI3K/Akt/mTOR signaling is one of the most frequent events in human cancer, especially in RCC and serves to disconnect the control of cell growth, survival and metabolism from exogenous growth stimuli (20). Hence, we speculated that GLUD1 suppressed ccRCC tumorigenesis and development by inhibiting PI3K/Akt/mTOR pathway. Western blotting results revealed that GLUD1 overexpression decreased the activation levels of Akt and mTOR in RCC cells (**Figure 5D**). These results indicate that the low level of GLUD1 promotes ccRCC tumorigenesis and development by activating PI3K/Akt/mTOR pathway.

### GLUD1 level is negatively associated with immunosuppressive microenvironment in ccRCC

**Figure 6:**
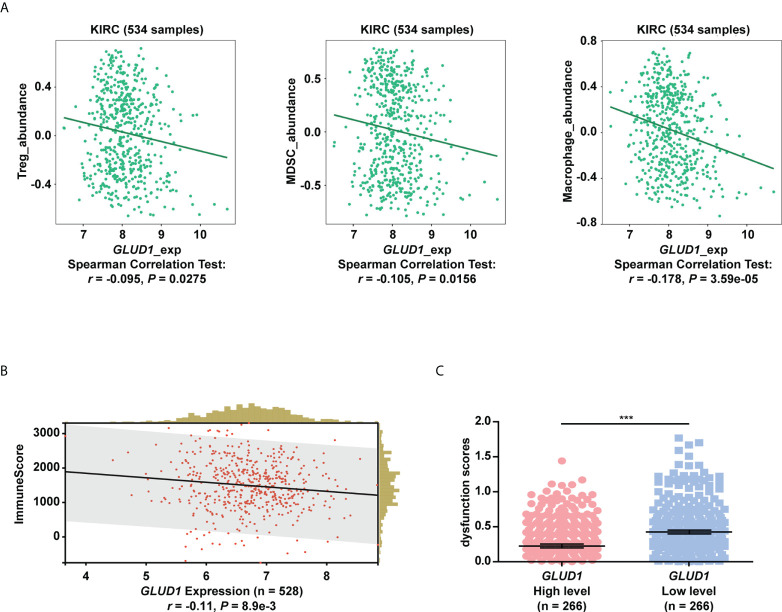
GLUD1 level is negatively associated with the ccRCC immunosuppressive microenvironment. **(A)** Correlation between GLUD1 level and immunosuppressive cell infiltration score in ccRCC patients. **(B)** Correlation of GLUD1 with immune cell infiltration in ccRCC. **(C)** The correlation between GLUD1 level and T cell dysfunction score by TIDE analysis. ***P < 0.001.

The above results showed that low levels of GLUD1 altered the metabolism of ccRCC cells by activating the mTOR pathway. Altered metabolic pathways, especially mTOR pathway in tumors play key roles in tumor growth and immnosuppressive acidic tumor environment (21). The suppressive immune microenvironment (TIME) reprograms immune cell behavior by altering the cellular machinery and nutrient supply of immune cells, thereby limiting antitumor function (21). We therefore assessed the correlation of GLUD1 expression with tumor immune microenvironment. The bioinformatics analysis results showed that *GLUD1* level in ccRCC cells was negatively related with the abundance of immunosuppressive cells (Treg cells, MDSCs and M2 macrophages) (**Figure 6A**), indicating that *GLUD1* low level was correlated with the TIME in ccRCC patients. In addition, GLUD1 low level was also related with the infiltration of other immune cells, including T cells (**Figure 6B**). If these T cells were in functional state, they would kill cancer cells. However, TIDE analysis result revealed that low *GLUD1* levels correlated with higher dysfunction scores of T cells (**Figure 6C**), suggesting that patients with low *GLUD1* levels and more immune cell infiltration tend to have a stronger signature of T cell dysfunction, which may impair the ability of cytotoxic T cells to kill cancer cells. Therefore, GLUD1 downregulation may contribute to the TIME by enhancing dysfunctional immune cell infiltration.

## Discussion

In this study, we described GLUD1 as a novel tumor suppressor in ccRCC, which was different from tumor-promoting role in other cancers. Moreover, we found that the low level of GLUD1 was correlated with the poor prognosis and low sensitivity to TKIs therapy for ccRCC patients. GLUD1 might be helpful for identifying patients who need individualized therapies to improve their clinical outcomes.

GLUD1 was reported to be highly expressed and play the tumor-promoting roles in breast cancer, gastric cancer and lung cancer (12, 22, 23). GLUD1 promoted breast cancer growth through accelerating metabolic recycling of ammonia (22). GLUD1 enhanced gastric cancer cell proliferation, migration and invasion by activating the Notch signaling pathway (12). GLUD1 regulated redox homeostasis to promote lung cancer cell proliferation (23). However, in highly proliferative breast tumors, GLUD1 was also reported to be expressed in low level (14), indicating that the controversial role of GLUD1 in breast cancer. In ccRCC, GLUD1 level was downregulated and acted as tumor suppressor and might be a therapeutic target for ccRCC.

GLUD1 protein level was reported to be downregulated *via* ubiquitination and degradation. Detailly, under conditions of amino acid deprivation, GLUD1 translocated from mitochondria to the cytoplasm, where it became ubiquitinated and degraded *via* the E3 ligase RNF213 (24). Our study found that GLUD1 level was downregulated in ccRCC tissues not only in protein level, but also in mRNA level. We also found that the upregulation of methylation level in the *GLUD1* promoter might be responsible for the downregulation of GLUD1 in ccRCC. This is a new mechanism of GLUD1 downregulation in ccRCC. By targeting GLUD1 methylation to regulate GLUD1 level might also be a therapeutic strategy for ccRCC.

Glutamine metabolism stimulates several signaling pathways that promote cell growth and proliferation. Of which, the activation of mTOR, a key signaling node regulating protein translation, cell growth and autophagy (25) is the most important event for glutamine metabolism. That glutamine was taken up in exchange for essential amino acids (EAA) is the rate-limiting step in mTOR activation of cancer cells (25, 26). α-KG from glutamine catabolism is critical for mTOR activation in cervical and osteosarcoma cancer cell lines (27). Hyperactivation of PI3K/Akt/mTOR signaling is critical for RCC cell proliferation, survival, migration and metastasis as well as angiogenesis and therapy resistance (28–31). In this study, we found a novel mechanism by which glutamine metabolism activated PI3K/Akt/mTOR in ccRCC-the downregulation of GLUD1 in glutamine metabolism pathway. Further, GLUD1 overexpression suppressed RCC cell proliferation and migration by inhibiting the PI3K/Akt/mTOR pathway activation.

In addition, GLUD1 was co-expressed with various metabolism-related genes in ccRCC. GLUD2 was downregulated in glioblastoma, and GLUD2 inhibited glioblastoma progression by promoting cell cycle arrest and leading to mitochondrial dysfunction (32). Rab21 was downregulated in ovarian cancer, which led to cytokinesis failure and induced aneuploidy, further underwent malignant transformation and tumorigenicity (33). NOD1 was highly expressed in colorectal cancer, and mediated the adhesion, migration and metastasis of colorectal cancer cells through the p38 MAPK pathway (34). DNMT3A was upregulated in liver cancer, and DNMT3A-mediated promoter hypermethylation inactivated multiple tumor suppressor genes, thus promoting liver cancer cell proliferation and colony formation (35). When we constructed the PPI network using GLDU1-related DEGs, GLUD1 was one of the hub genes of these co-expressed or related genes. This confirmed that GLUD1 played a key role in ccRCC development.

ccRCC has been reported to be a highly immunogenic malignancy that has been shown to be infiltrated by a large amount of immunocytes, including macrophages, NK cells, and T cells (36). Several studies have shown that the abundance of tumor-infiltrating lymphocytes and CD8^+^ T cells is inversely associated with prognosis of ccRCC patients (37, 38). As for immune cell infiltration and immunotherapy, the use of anti-PD-1 or anti-PD-L1 therapy in ccRCC patients have been reported. Nivolumab is considered as a standard care strategy for advanced ccRCC and is widely used in clinical trials (39). Depleted CD8^+^ T cells and M2-like macrophages co-occur in advanced disease and express ligands and receptors that support T-cell dysfunction and M2-like polarization, an immune dysfunctional circuit leading to poor prognosis (40). In the current study, we revealed an inverse correlation between *GLUD1* level and immune cell infiltration, and GLUD1 promotes mTOR pathway activation. mTOR pathway activation results in the upregulation of HIF-1α and the increase in glycolysis and lactate production (41). An acidic TME favors immunosuppression, reduces response to immunotherapy, and stabilizes immunosuppressive cells (21). Meanwhile, the expression of PD-L1 protein is dependent on active Akt-mTOR signaling. The interaction of PD-L1 and PD-1 induces differentiation of naïve CD4^+^ T cells into Tregs and maintains Treg-suppressive functions. PD-L1 can also act as a receptor by sending reverse signals to limit tumor cell apoptosis (42). Therefore, utilizing drugs targeting GLUD1 metabolism may synergistically enhance renal cancer immunotherapy through metabolic reprogramming of the TME.

Taken together, this study revealed that GLUD1 might predict the prognosis of ccRCC patients, especially advanced ccRCC patients. GLUD1 suppressed the occurrence and development of ccRCC by inhibiting the PI3K/Akt/mTOR pathway. GLUD1 may be a potential therapeutic target for ccRCC, and a combination of GLUD1 targeted therapy and immunotherapy may provide better therapeutic efficacy for ccRCC.

## Data availability statement

Publicly available datasets were analyzed in this study. This data can be found here: https://www.ncbi.nlm.nih.gov/geo/query/acc.cgi?acc=GSE53757 and https://www.cbioportal.org/study/summary?id=kirc_tcga.

## Ethics statement

The studies involving human participants were reviewed and approved by the Research Ethics Board of Affiliated Beijing Friendship Hospital. The patients/participants provided their written informed consent to participate in this study.

## Author contributions

LW participated in tissue collection, analysis of data, development of methodology and designing the experiment. ZF conceived the experiment, carried out the experiments and analyzed the data. PG performed the experiments and wrote the manuscript. JZ designed the experiment and reviewed the manuscript. All authors discussed the results, revised and commented on the manuscript.

## Funding

This work was supported by the National Natural Science Foundation of the People’s Republic of China (Nos. 81974415, 82172923).

## Conflict of interest

The authors declare that the research was conducted in the absence of any commercial or financial relationships that could be construed as a potential conflict of interest.

## Publisher’s note

All claims expressed in this article are solely those of the authors and do not necessarily represent those of their affiliated organizations, or those of the publisher, the editors and the reviewers. Any product that may be evaluated in this article, or claim that may be made by its manufacturer, is not guaranteed or endorsed by the publisher.
