# Supplementary material for: GLUD1 suppresses renal tumorigenesis and development via inhibiting PI3K/Akt/mTOR pathway
Source: Front Oncol. 2022 Sep 20;12:975517. doi: 10.3389/fonc.2022.975517 (PMC9530280; doi:10.3389/fonc.2022.975517)
Supplement: Supplementary file 1 [file DataSheet_1.docx]

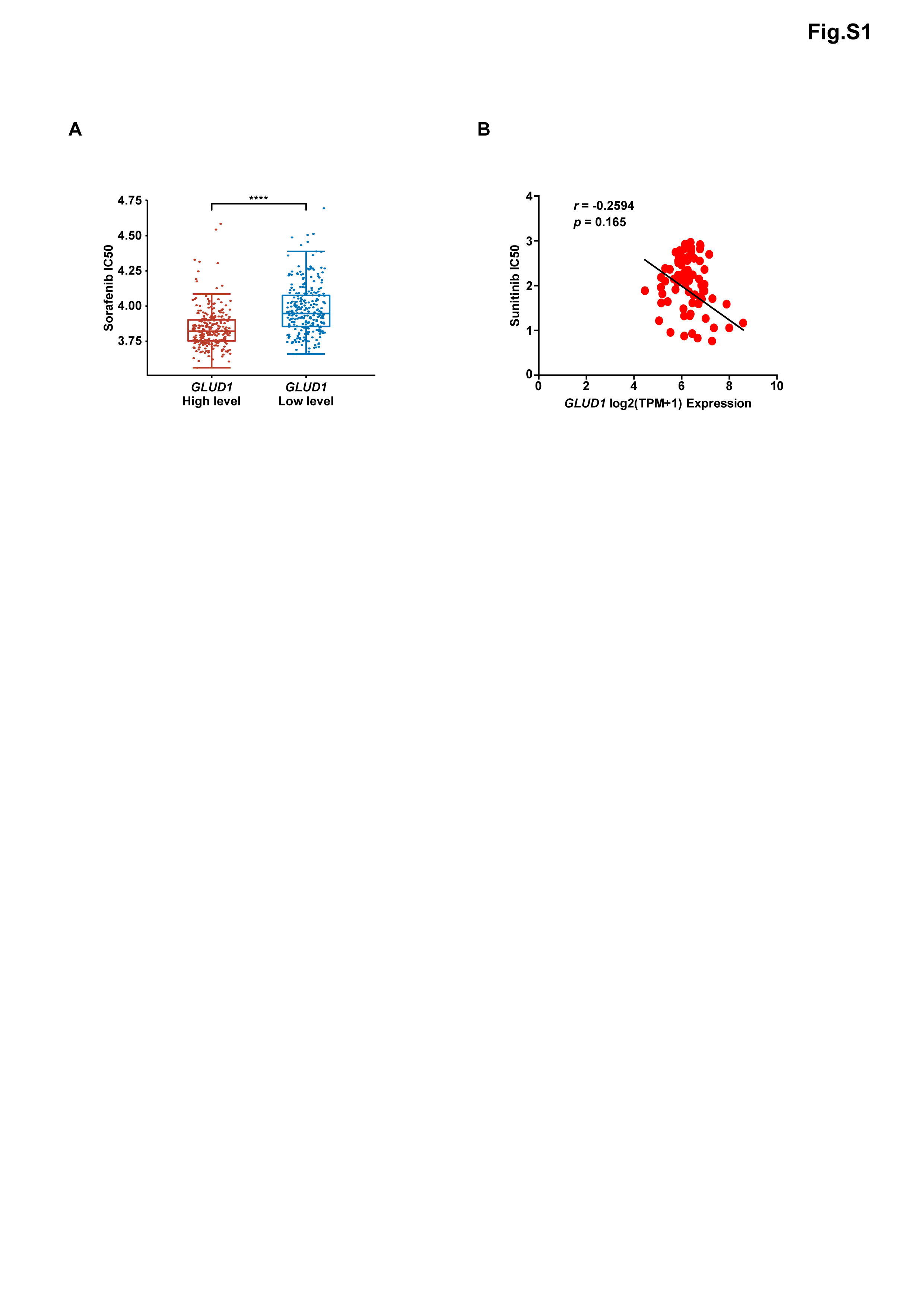


**Figure S1:** *GLUD1* expression levels correlate with TKIs drug sensitivity. **(A)** Based on the Genomics of Cancer Drug Sensitivity (GDSC) database (www.cancerrxgene.org), the predicted half maximal inhibitory concentration (IC50) of GLUD1 expression levels in response to Sorafenib treatment. The abscissa represents different groups of samples, and the ordinate represents the distribution of the IC50 score. **(B)** Correlation analysis of *GLUD1* expression and Sunitinib IC50 score based on CellMiner database (https://discover.nci.nih.gov/cellminer/home.do).


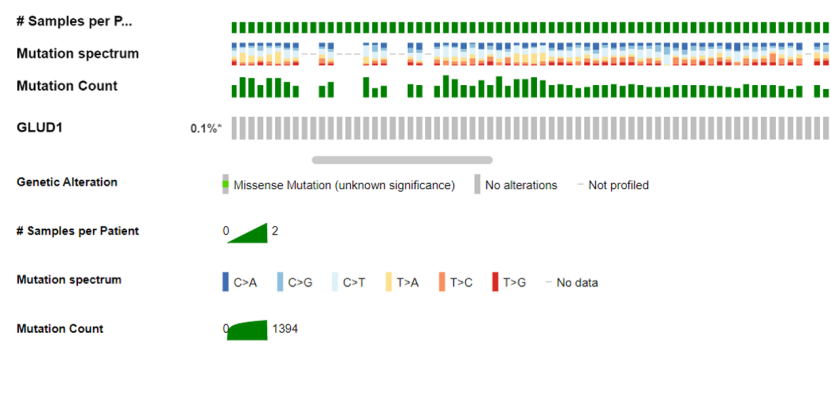


**Figure S2:** *GLUD1* gene has low frequency in mutation. OncoPrint from cBioPortal showed that *GLUD1* gene changed only in 0.1% of ccRCC cases.


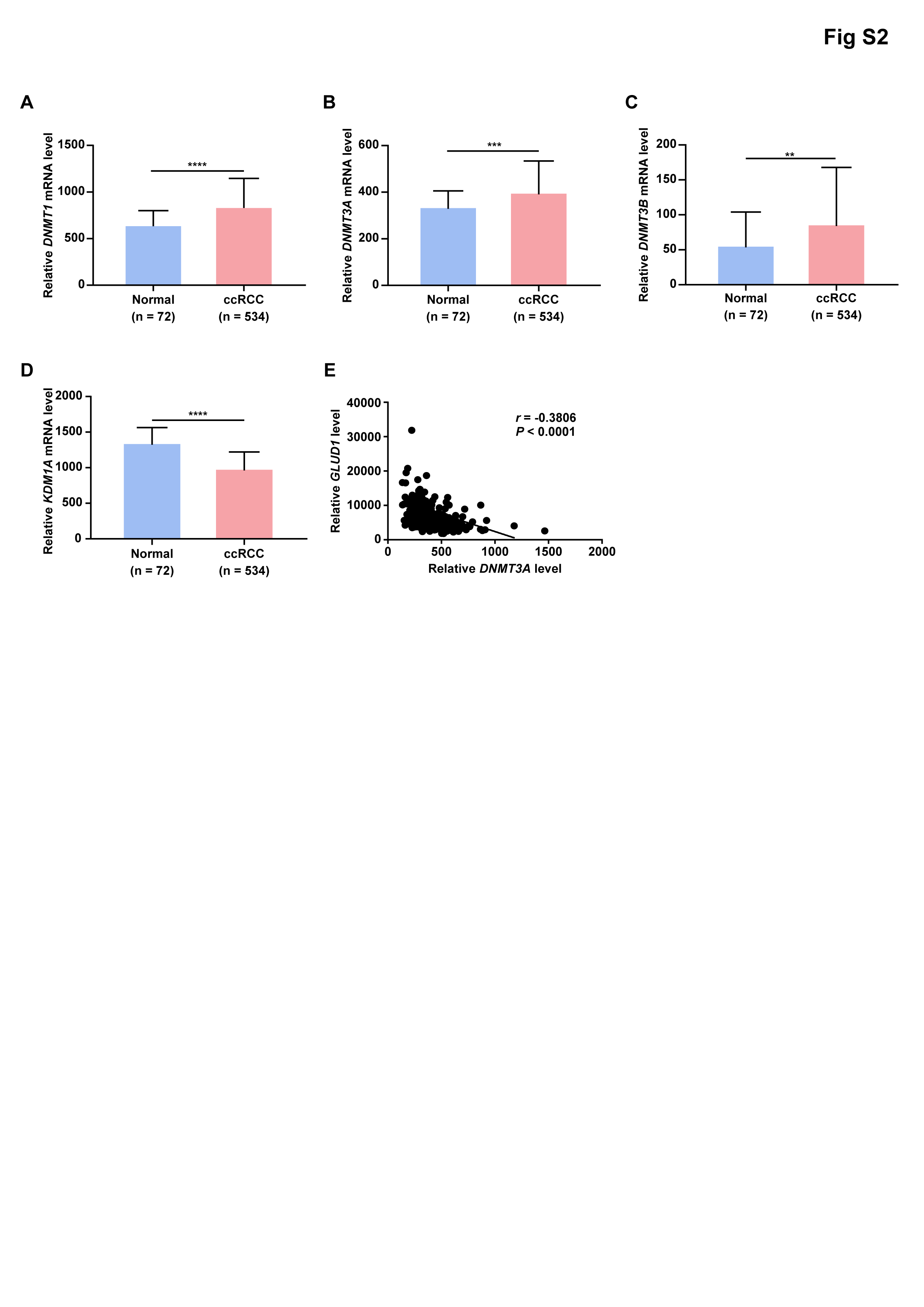


**Figure S3:** DNMT3A may play a crucial role in regulating the methylation level of *GLUD1* promoter in ccRCC. **(A-D)** Expression level analyses of methylation-related writers and erasers showed *DNMT1*, *DNMT3A*, *DNMT3B* and *KDM1A* mRNA levels were differentially expressed between adjacent normal tissues and ccRCC tissues based on TCGA_KIRC data. **(E)** Based on TCGA data, correlation between methylation level in *GLUD1* promoter and *DNMT3A* mRNA level was analyzed.
